# Amiodarone in the treatment of atrial fibrillation of patients with rheumatic heart disease after valve replacement

**DOI:** 10.12669/pjms.35.4.1298

**Published:** 2019

**Authors:** Kebiao Chen, Li Qin, Xin Lu, Tao Xia, Qing Gu

**Affiliations:** 1 Kebiao Chen, Department of Cardiovascular Surgery, Taian Central Hospital, Shandong, 271000, China; 2 Li Qin, Department of Cardiovascular Surgery, Taian Central Hospital, Shandong, 271000, China; 3 Xin Lu, Department of Cardiovascular Surgery, Taian Central Hospital, Shandong, 271000, China; 4 Tao Xia Department of Cardiovascular Surgery, Taian Central Hospital, Shandong, 271000, China; 5 Qing Gu Department of Cardiovascular Surgery, Taian Central Hospital, Shandong, 271000, China

**Keywords:** Amiodarone, Rheumatic heart disease, Atrial fibrillation

## Abstract

**Objectives:**

To explore the efficacy of amiodarone in the treatment of atrial fibrillation for patients with rheumatic heart disease after valve replacement.

**Methods:**

Eighty-six patients with rheumatic heart disease who were hospitalized between June 2016 and June 2017 and developed atrial fibrillation after valvular heart valve replacement were randomly divided into a control group and an observation group, 42 cases in each group. The control group was treated with routine medical treatment, while the observation group was given amiodarone on the basis of routine treatment. The cardiac function of the two groups were observed and recorded. Postoperative atrial fibrillation conversion rate, sinus rhythm maintenance rate, intensive care unit (ICU) monitoring time and hospital stay were compared between the two groups.

**Results:**

Compared with the control group, the improvement of cardiac function indexes of the observation group was better, and the difference was statistically significant (P<0.05). The atrial fibrillation conversion rate and the maintenance rate of sinus rhythm of the observation group were 76.2% and 47.6% respectively, which were significantly higher than 57.1% and 33.3% of the control group; the differences had statistical significance (P<0.05). The ICU monitoring time and hospitalization time of the patients in the observation group were (1.69±0.91) d and (10.24±1.11) d respectively, which were significantly shorter than (2.83±0.95) d and (14.07±1.17) d in the control group (P<0.05); the differences were statistically significant (P<0.05).

**Conclusion:**

Amiodarone can effectively treat valve replacement associated atrial fibrillation of patients with rheumatic heart disease. It can significantly improve the heart function, prevent the recurrence of atrial fibrillation, maintain sinus rhythm after operation, and shorten the time of ICU monitoring and hospitalization.

## INTRODUCTION

Atrial fibrillation is the most common persistent arrhythmia in clinical practice.^1^,^2^ The incidence of atrial fibrillation after valve replacement is as high as 65% among patients with rheumatic heart disease. The incidence of atrial fibrillation after simple mitral valve replacement and aortic valve replacement is 35.1% and 36.2% respectively, and the incidence of atrial fibrillation after mitral valve replacement combined with aortic valve replacement can be as high as 55.4%.^3^ In addition, postoperative atrial fibrillation generally has a low conversion rate, and the sinus rhythm is poor after conversion, which may have adverse effects on patients, cause hemodynamic disorders and even cardiogenic shock, and increase risks of stroke, heart failure and hospitalization mortality.^4^,^5^ Therefore, how to maintain sinus rhythm effectively after valve replacement, prevent recurrence of atrial fibrillation, avoid complications such as stroke and heart failure, and effectively improve the quality of life of patients have become important problems in cardio-thoracic surgery.

A recent study shows that amiodarone has a unique role in preventing postoperative atrial fibrillation.^6^ Roy D et al. found that amiodarone was safe and effective in the conversion and maintenance of sinus rhythm and can reverse atrial fibrillation by extending atrial effective refractory period and non-competitive antiepinephrine effect.^7^ Amiodarone has been widely used in the conversion of atrial fibrillation, and its conversion efficiency is as high as 55%.^8^ This study investigated the effect of amiodarone on atrial fibrillation of patients with valvular replacement associated rheumatic heart disease, aiming to provide a reference for the treatment of valve replacement associated atrial fibrillation.

## METHODS

### Research Data

In the study, eight-four patients with rheumatic heart disease who were admitted into our hospital between June 2016 and June 2017 and had valvular replacement associated rheumatic heart disease were selected. Patients who underwent elective valvular replacement, had rheumatic heart disease in combination with continuous atrial fibrillation, had no other types of arhythmia, had cardiac function no higher than grade III,^9^ satisfied the application indication of amiodarone, had normal level of electrolyte and acidity and alkalinity, and had heart rate lower than 70 times/min were included. The patients were divided into a control group and an observation group according to the random digital table method, 42 cases each group. The gender composition, average age, left atrial diameter (LAD), duration of atrial fibrillation, heart function classification of (New York Heart Association (NYHA), cardio-thoracic ratio and left ventricular ejection fraction (LVEF) of the two groups had no remarkable differences (P>0.05); hence the results were comparable (Table-I). This study was approved by the ethics committee of our hospital. All the patients signed informed consent.

**Table I:** Baseline characteristics of study population

Group	Observation group	Control group
Male/female	22/20	21/21
Age (year)	65.36±10.77	65.45±10.82
Duration of atrial fibrillation (month)	30.96±14.93	31.06±15.02
LAD (mm)	45.87±3.95	47.06±3.35
LVEF(%)	46.7±4.32	47.5±4.03
Cardio-thoracic ratio	0.54±0.07	0.56±0.08
NYHA	2.50±0.51	2.58±0.50

### Method

Patients in the control group received routine postoperative drug treatment including oral administration of drugs for diuresis, anticoagulation and routine application of antibiotics. On the basis of routine treatment, the observation group was additionally treated with amiodarone (Sanofi Minsheng Pharmaceutical Co., Ltd.; batch number: 7h90042). On the day of the operation, a micro infusion pump was used to pump 600 mg of amiodarone injection at a speed of 50 mg/h, for 12 hours. On the postoperative 1^st^ day, the patients orally took amiodarone after recovery of diet, 3 times a day; one week later, the patients took it twice each day; one week later, the patients took it once daily. The course of treatment was one month.

### Observational Indexes

Cardiac function indexes including peak velocity of peak A (VA), dispersion of wave P (Pd), the maximum time limit of wave P (Pmax), the maximum volume of the left atrium (LAV_max_) and ventricular rate were observed after treatment.

The conversion rate of atrial fibrillation, maintenance rate of sinus rhythm, ICU monitoring time and hospitalization time of the two groups were also observed after treatment.

### Statistical Processing

Data were analyzed using SPSS 21.0. Measurement data were expressed as mean±standard deviation (SD), Comparison between groups was performed using *t* test. Enumeration data were processed using Chi-square test. Difference was thought statistically significant if the value of p was smaller than 0.05.

## RESULTS

### Cardiac Function of the Two Groups

The improvement of the cardiac function indexes of the observation group was better than that in the control group after treatment, and the difference was statistically significant (P<0.05, Table-II).

**Table II:** Comparison of different cardiac function indexes (mean±SD).

Group	Observation group	Control group	t	P
VA(cm/s)	63.41±6.65	58.28±6.76	3.812	<0.05
Pd(ms)	45.48±6.09	50.02±6.24	3.701	<0.05
P_max_ (ms)	112.03±11.26	121.51±10.99	4.278	<0.05
LAV_max_ (cm3)	19.10±5.34	26.38±5.61	6.635	<0.05
Heart rate(times/mi)	80.41±3.37	88.89±3.63	12.124	<0.05

### The conversion of atrial fibrillation and sinus rhythm of the two groups

After treatment, the maintenance rate of sinus rhythm and conversion rate of atrial fibrillation were obviously superior to those of the control group, and the differences between the two groups were statistically significant (P<0.05, Table-III).

**Table III:** The conversion of atrial fibrillation and maintenance of sinus rhythm [n(%)].

Group	Observation group	Control group	X^2^	P
Conversion rate of atrial fibrillation	32(76.2)	24(57.1)	4.483	<0.05
Maintenance rate of sinus rhythm	20(47.6)	14(33.3)	4.229	<0.05

### ICU monitoring time and hospitalization time of the two groups

After treatment, the ICU monitoring time of the observation group was significantly shorter than that of the control group ((1.69±0.91) d vs. (2.83±0.95) d), and the difference was statistically significant (t=3.841, P<0.05). The hospitalization time of the observation group was significantly shorter than that of the control group ((10.24±1.11) d vs. 14.07±1.17) d, and the difference was statistically significant (t=7.254, P<0.05, Table-IV).

**Table IV:** The comparison of ICU monitoring time and hospitalization time (mean±SD).

Group	Observation group	Control group	t	P
ICU monitoring time(d)	1.69±0.91	2.83±0.95	3.841	<0.05
Hospitalization time(d)	10.24±1.13	14.07±1.17	7.254	<0.05

## DISCUSSION

Rheumatic heart disease is induced by rheumatic fever which involves heart valve. With the improvement of living standard, the incidence of rheumatic heart disease is increasing.^10^ Valve replacement has become the main therapy for rheumatic heart disease.^11^ Patients often have atrial fibrillation after undergoing rheumatic heart valve replacement. Patients with mild atrial fibrillation may have disturbance of hemodynamics in the perioperative period, and patients with severe atrial fibrillation may have severe heart failure, stroke, and cardiogenic shock or even die. Studies found that improving the conversion rate of atrial fibrillation and maintaining sinus rhythm was the key after rheumatic heart disease associated valve replacement.^12^,^13^

Amiodarone which is the third class of antiarrhythmic drug is a β and α adrenergic receptor inhibitor. Its bioavailability is about 50%. It is mainly distributed in the organs which were rich in fat, such as lymph gland, liver, kidney and lung. 34% of amiodarone in plasma can be combined with beta lipoprotein, and 62% can be combined with albumin; moreover it can be eliminated through metabolism in the lung. The half-life of the drug is longer, 15 d to 28 d. It can be absorbed by tissue after 4.6 h when the dose was 0.8 g. The blood concentration of amiodarone can reach the peak at the 4^th^ to 6^th^ h, and a low blood concentration can still be detected even half a year after withdrawal.^14^,^15^ Its main pharmacological function is to eliminate re-entrant excitation, inhibit the rapid sodium ion internal flow of the atrial and myocardial conduction fibers and reducing sinus-node self-activity through prolonging the action potential and effective refractory period of the myocardial tissue of patients.^16^ In addition, amiodarone has four kinds of antiarrhythmic functions. It can inhibit the potassium channel and has no influence on the height of resting membrane potential and action potential. Although there is a slight negative inotropic effect in static injection, it usually will not inhibit the left ventricular function. Moreover it has direct dilation effect on the coronary artery and the surrounding blood vessels, which is beneficial to improve the postoperative haemodynamics of patients.^17^,^18^ Compared with other antiarrhythmic drugs, amiodarone has higher conversion rate and better sinus rhythm maintenance.^19^ In this study, the conversion rate of atrial fibrillation and maintenance rate of sinus rhythm of the observation group were 76.2% and 47.6% respectively, which were significantly higher than those of the control group, and the results were similar to the previous research results.^20^,^21^ In addition, rate of administration ways of amiodarone can produce different effects. Intravenous administration makes amiodarone take effect late, while oral administration is the opposite. The dosage regimen can be formulated according to the postoperative condition of patients. Amiodarone has few side effects and is less likely to induce complications such as embolism after cardioversion, which is beneficial to the recovery of patients. In this study, the ICU monitoring time and time of hospitalization of the observation group was (1.69±0.91) d and (10.24±1.13) d respectively, which were significantly shorter than that of the control group, which was similar to the research results of Zhang et al.^22^

In addition, amiodarone can also reduce peripheral vascular resistance and increase myocardial blood pumping, which not only maintain the volume of blood transfusion, but also reduce heart rate. Amiodarone can maintain the oxygen consumption of the myocardium, improve the function of diastole and contraction of the myocardium through expanding the coronary artery, increasing the blood supply of the myocardium and improve the working state of the heart.^23^ Wang et al. found that amiodarone could prolong the interval between PR and QT and improve the structure and function of the myocardium on the basis of lowering blood pressure and slowing down the heart rate.^24^ Jie et al. also found that amiodarone could significantly improve the cardiac recovery of patients with atrial fibrillation.^25^ The results of this study also showed that the indexes of cardiac function in the observation group improved significantly, which was similar to the above research result.

## CONCLUSION

For patients with rheumatic heart disease who developed valve replacement associated atrial fibrillation, amiodarone can improve the cardiac function, prevent the recurrence of atrial fibrillation, maintain sinus rhythm for a long time and accelerate recovery. Although amiodarone is effective in the treatment of postoperative atrial fibrillation of patients with rheumatic heart disease, further studies are still needed. For example, whether use of amiodarone at a smaller dosage or earlier use of amiodarone can achieve better results and how sinus rhythm will change after conversion during long-time follow up remains to be verified through longer clinical follow up.

### Authors’ Contribution

**KBC & LQ:** Study design, data collection and analysis.

**KBC, XL & TX:** Manuscript preparation, drafting and revising.

**KBC & QG:** Review and final approval of manuscript.
